# 

**Published:** 1910-12

**Authors:** 


					﻿PUBLISHED MONTHLY.
SUBSCRIPTION $1.00
ATLANTA
JOURNAL-RECORD
OF MEDICINE Fi
i Atlanta Medical and Surgical Journal, Established	y
and Southern Medical Record, Established 1870\ "L-------|3Wh- rvuf
Vol. LVI.
Atlanta, Ga., December, 1910.
No. 9
				

